# A Three Month Assessment of Anthropogenic Litter Levels on the Illinois River, Tahlequah, Oklahoma

**DOI:** 10.17912/micropub.biology.001363

**Published:** 2024-11-26

**Authors:** Emma C. Mills, Katherine M. Wollman, Elizabeth F. Waring

**Affiliations:** 1 Department of Biological Sciences, Northeastern State University, Tahlequah, Oklahoma, US; 2 Department of Ecosystems and Watershed Management, Grand River Dam Authority, Langley, OK, US

## Abstract

Anthropogenic litter is one of the most important factors that influence recreation users and their activities because of its correlation to the river and environmental health. We monitored pollution levels on the Illinois river, near Tahlequah, OK for three months and surveyed the publics opinion on the issue. Our goal was to get this data to local and state management agencies to management practices to keep the scenic Illinois River clean. We found aluminum cans in the most abundant, while survey participants said cigarette butts were of highest observable abundance. A total of 170 people took the survey, and many of them want to see change on the river. Change may have to come from cooperation with commercial float operators.

**Figure 1. Results of River User Survey:**
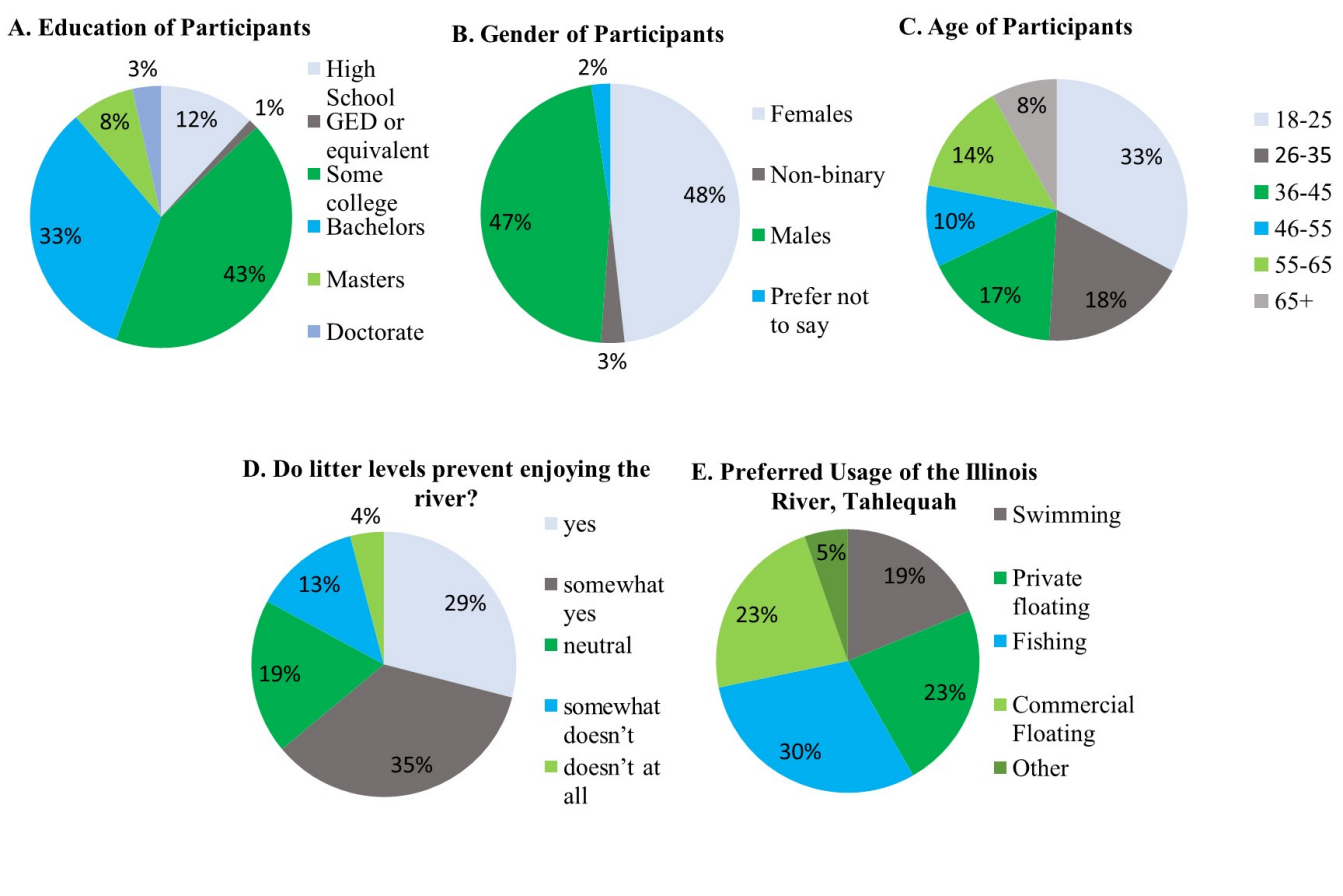
**A** . Education of Participants: High School (20), GED or equivalent (2), Some college (72), Bachelors (56), Masters (13), Doctorate (6); N=169. **B** . Gender of Participants: Females (82), Males (79), Non-binary (5), Prefer not to say (4); N=170. **C** . Age of Participants (years old): 18-25 yo (52), 26-35 yo (29), 36-45 yo (27), 46-55 yo (16), 55-65 yo (22), and 65+ yo (13); N=159. **D** . Do little levels prevent you from enjoying time at the river?: Yes (49), Somewhat yes (59), Neutral (31), Somewhat doesn’t (22), Doesn’t at all (7); N=168. **E** . Preferred usage of the Illinois River: Swimming (32), Floating (39), Fishing (51), Commercial floating (39), Other (8); N=169

## Description

The Illinois River of Tahlequah Oklahoma is a frequent hotspot for recreation in the summer for locals and non-locals alike (Meo et al. 2002) . Though responsible for an influx in economic growth, humans leave a heavy footprint on the Illinois River. With growing attraction, new management practices are needed in Tahlequah and other regulated waters in the United States (Cowger et al. 2019) . Anthropogenic litter is one of the most important factors that influence recreation users and their activities because of its correlation to the river and environmental health (Adam 2021) .

There is a positive relationship between human population density and the amount of anthropogenic litter found in other freshwater systems (HoelIein et al. 2015, Vincent et al. 2017). Due to Tahlequah’s economic reliance on the Illinois River in the summer months, it is a growing concern for how the river is perceived and treated. To assess this, we monitored anthropogenic litter levels on the Illinois river, Tahlequah for three months and surveyed the publics opinion on the issue. This was done by collecting the litter, sorting/weighing it, and dispersing an IRB-approved survey to the Tahlequah area and social media. We used the survey to access how people perceive the river and how it really is. Our goal is to allow for the recreational users and stakeholders of the Illinois River to use this data and research for better management practices to keep the the scenic Illinois River clean.

The survey received a total of 170 responses ( Figure 1A -E). It concluded that 65% of all participants are at least somewhat bothered by litter levels on the Illinois River, and suggestions were given with 85% of completed surveys. Most participants (45.8%) use the river for commercial/private floating, while some use it for fishing (30%) and swimming (18.8%). Of all commercial float participants, only 48% were offered/issued a trash bag. Litter was said to be more commonly seen on beaches at 79.4% than water at 20.6%. Only 13.6% of participants admitted to ever littering. These results are similar to those reported by Uogintė et al. (2024) in Lithuanian streams. Showing that the issues with anthropogenic litter are a worldwide issue.

**Table:** 

Litter Type	Reported Common Litter Types from Survey	Number of Items Collected
Metals	139	2377
Plastic	138	1066
Wrappers	39	919
Personal Items	96	477
Miscellaneous	22	279
Glass	33	92
Styrofoam	38	74

**Table 1:** Table comparing reported most common litter type from survey takers and number of counted items of litter collected between May and August in 2022.

We found the highest abundance in aluminum, plastic, then wrappers (Table 1). Over the three months of collection, we sorted a total of 5,284 pieces of anthropogenic litter items weighing 260.7kg. In the least abundance was glass and Styrofoam. Glass and Styrofoam were expected to be of the lowest abundance of anthropogenic litter reported by survey participants. In similarity, they were found in low numbers while collecting and sorting anthropogenic litter. This is to be expected as currently, glass and Styrofoam are banned on the Illinois River due to Oklahoma Administrative Code 300:40-4-3 - Certain containers prohibited. These results are reflective of what would be expected in the summer months. In the fall and winter, the float operators shut down for the year and the number of users of the river decreases substantially. We would expected these numbers to be small in the winter, but as litter collection crew only works in the summer months.

Due to the recreational usage of the Illinois River, it is no surprise aluminum cans were of the highest abundance (Table 1). The river is lined with operators outfitting high volumes of floaters during the summer months, as this is a preferred use of the water (Fig 1E). Floaters often bring along soft and hard canned drinks that are frequently left behind to alter the ecosystem. To reduce litter, floaters are supposed to be offered or given a small plastic bag to contain their trash, but only 48% of all commercial floaters were issued one.

Litter on beaches is more apparent to survey participants than in the water (Table 1). This is possibly due to the activities participated in while on beaches rather than just swimming. Changes in river flow, precipitation, and holidays affect litter levels and distribution changing participants' perceptions and our abilities to accurately collect all litter.

Over half of all participants were bothered to some extent by the levels of litter on the Illinois river. It is known that with an increase in education comes an increase in awareness and sensitivity of beach litter (Adam 2021) . Approximately 87% of participants had some college education or above. Education of the public to promote understanding and desire for change is required to address water quality issues (Wagner 2021) . Previous studies show Oklahoma's lack of concern for clean water and the associated knowledge. A little under half of Oklahomans are ignorant of what pollutants can even cause water quality issues (Wagner et al. 2021) .

Even though the river is overrun with anthropogenic litter, only a few participants admitted to ever littering. People are victims of litter, but they are also the polluters themselves (Adam 2021) . We expected the surveys to report more people having once littered, this could be due to the wording of the question or sample size. It has been observed that the general population does not jump at the opportunity to participate in scientific studies (Wagner et al. 2021) or our sample size would have possibly been larger.

## Methods

• **Cleaning and gathering of all anthropogenic litter** : We navigated a section of the Illinois River nearest Tahlequah, OK, and collected anthropogenic litter from the water and shoreline using deep canoes, trash grippers, trash bags, and 10-gallon buckets. This was done in sections once a month to three times a week by the Grand River Dam Authority (GRDA) float crew and our lab members. The sections that anthropogenic litter is collected from are from Arkansas/Oklahoma state line to Round Hollow Public Access Area (PAA) which is ~27.7 miles. The second section is from Chewey Bridge to No Head Hollow PAA which is ~23.6 miles. The last section that anthropogenic litter was collected from was from Comb’s Bridge to and including the confluence of Barren Fork Creek which is ~36.1 miles.

• **Sorting and weighing the litter** : After the bagged litter was transferred to the GRDA facility, the bags were weighed individually using a fish scale. The trash was sorted on tarps into the following categories: plastic, glass, tin, aluminum, personal items, Styrofoam, wrappers, and misc. These categories were then weighed and counted. The sorted trash was disposed of at a local landfill except for the aluminum items were sent to a recycling plant.

• **Assess users of the Illinois River:** Concurrently we created a 15-question Google form survey to access the peoples’ perceptions, habits, and knowledge of the Illinois River. This included questions on demographics, opinions, and open-ended writing. The survey was approved through Northeastern State University’s Institutional review board (IRB # 22-059).
